# Relationship of the Pelvic-Trochanteric Index with greater trochanteric pain syndrome

**DOI:** 10.6061/clinics/2021/e3312

**Published:** 2021-11-17

**Authors:** Leandro Emílio Nascimento Santos, Túlio Pinho Navarro, Carla Jorge Machado, Henrique Antônio Berwanger de Amorim Cabrita, Robinson Esteves Pires, Leonardo Brandão Figueiredo, Henrique Melo Campos Gurgel, Rudolf Moreira Pfeilsticker, Helvécio Grandinetti, Amanda Damasceno de Souza, Marco Antônio Percope de Andrade

**Affiliations:** I Hospital Felício Rocho, Belo Horizonte, MG, BR.; II Universidade Federal de Minas Gerais, Belo Horizonte, MG, BR.; III Instituto VITA, Sao Paulo, SP, BR.; IV Hospital das Clinicas HCFMUSP, Faculdade de Medicina, Universidade de Sao Paulo, Sao Paulo, SP, BR.

**Keywords:** Hip Injuries, Pelvis, Radiology, Tendinopathy, Waist-Hip Ratio

## Abstract

**OBJECTIVES:**

This study aimed to correlate a higher Pelvic-Trochanteric Index (PTI) with an increased varus of the femoral neck with greater trochanteric pain syndrome (GTPS). The secondary objective was to check whether the pelvic width changes with age.

**METHODS:**

A prospective study was conducted to compare female patients diagnosed with GTPS (case group) with asymptomatic female participants (control group) from March 2011 to June 2017. On an anteroposterior pelvic radiograph, lines were drawn by two radiologists, and the PTI (ratio of the distance between the greater trochanters and distance between the iliac crests) was defined and the femoral neck-shaft angle was measured.

**RESULTS:**

Data collected based on radiographs of 182 female patients (cases) and 150 female participants (controls) showed that the mean PTI was 1.09 (SD=0.01) in the case group and 1.07 (SD=0.01) in the control group (*p*<0.05), regardless of age. The distance between iliac crests increased with age (*p*<0.05) in symptomatic and asymptomatic individuals. It was also found that the mean femoral neck-shaft angle was 130.6° (SD=0.59) and progression of the varus angulation occurred with age in both groups, with a significance level of 5%.

**CONCLUSIONS:**

The PTI was higher in patients with GTPS. The femoral neck-shaft angle does not differ between individuals with and without GTPS; however, it does decrease with age. The pelvic width tends to increase with aging in symptomatic or asymptomatic individuals; therefore, the increase in the pelvic width and decrease in the femoral neck-shaft angle can be interpreted as normal in aging women, which could alter the biomechanics of the hips and pelvis.

## INTRODUCTION

Greater trochanteric pain syndrome (GTPS) or lateral peritrochanteric syndrome of the hip includes trochanteric bursitis, external snapping hip, and gluteus medius and minimus tendinopathies and tears (1). Symptoms of GTPS include pain exacerbated while walking or lying on the symptomatic side, sometimes radiating distally to the thigh, proximal iliotibial band pain (2), and snapping over the greater trochanter.

The prevalence of GTPS is 10%-20% in the general population, with an incidence of 1.8 per 1,000 people. It also occurs between the fourth and sixth decades of life (3). According to Williams et al. (4), the highest prevalence among women can be associated with differences in size, format, pelvic orientation, and its relation with the iliotibial band. Similarly, hip abductor tendon injuries, such as gluteus medius and minimus tendinopathies, are more common in women, possibly due to their increased pelvic width, as reported in 25% and 10% of middle-aged women and men, respectively (5-8).

Thus, a method that could determine this greater width of the pelvis and correlate it with GTPS would help hip surgeons to confirm the diagnosis or at least to use prophylactic therapeutic methods to prevent GTPS.

As a radiographic image guide, we postulated that the Pelvic-Trochanteric Index (PTI), defined as a higher ratio between the distance from the greater trochanter and the distance between the iliac crests, could be correlated with GTPS.

We hypothesized that the PTI and varus of the femoral neck would be higher in patients with GTPS than in asymptomatic patients. We also checked whether the pelvic width changed with age.

## MATERIALS AND METHODS

The study was approved by the Ethics in Research Committee of our hospital, under protocol CAAE:0012.0.240.000.11. This case-control study compared a group of female patients diagnosed with GTPS and a group of asymptomatic females matched for age and weight.

The case group included adult female outpatients (aged 18-90 years) who were diagnosed with recalcitrant GTPS based on criteria standardized by two independent hip surgeons (characteristic history, pain on palpation of the greater trochanter, external snapping hip, MRI with gluteus medius and/or minimus tears, and thocantheric bursitis or iliotibial band tears). Anteroposterior pelvic radiographs were collected from March 2011 to June 2017. Radiographs were obtained by trained X-ray technologists.

The control group included healthy female volunteers without a clinical diagnosis of GTPS, without lumbar or lower-extremity symptoms, who entered the trauma section of our hospital with upper-limb injuries, and who agreed to undergo a pelvic X-ray from November 2016 to June 2017. The risk of radiation for the volunteers was minimal, and they were protected with lead belts. The volunteers were healthy, had no recent radiological examinations, and all agreed to participate in the study.

Patients with any neurological, psychiatric, and/or cognitive deficit; previous fracture of the pelvis and lower limbs; previous surgery of the hip joint or femur; any hip disease other than GTPS (to the case group); morbid obesity (BMI>40 kg/m^2^); pregnancy; children and adolescents under 18 years of age; male sex; and those who did not sign the informed consent form were excluded from this study. To ensure that no participant with crossed symptoms would be part of this group, range of motion (ROM) and physical examinations were performed by two independent hip surgeons.

For statistical purposes, a 10% level was considered for type I errors, with 80% test power and 15% margin of error for differences among continuous measurements. As this was a case-control study, 120 patients were considered the minimum sample size, at a 1:1 ratio with the control group. Therefore, at least 240 individuals were included in the study.

A total of 182 patients with GTPS (pain on the greater trochanter, pain exacerbated while walking or lying down on the symptomatic side, sometimes extending distally to the thigh, and snapping over the greater trochanter that refers to the motion of the iliotibial band over the lateral aspect of the greater trochanter of the femur) were included in the study group. A total of 150 healthy female volunteers were enrolled in the control group, and all volunteers signed an informed consent form and underwent pelvic radiography with lead belt protection.

### Procedures

A 10% standard magnification anteroposterior pelvic radiography was performed. A distance of 1 m was maintained between the X-ray tube and the chassis, and the patient assumed the supine position with 20° internal rotation of the lower extremities and with both hallux touching each other.

**Figure 1:**
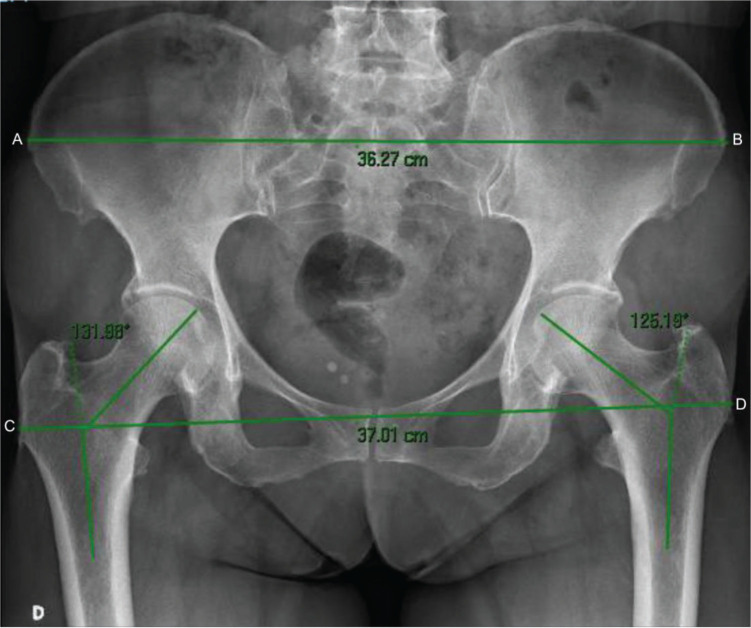
Distances between the greater trochanter and iliac crest and demonstration of the measurement of the femoral neck-shaft angle. AB, distance between the external lateral ends of the iliac crest; CD, distance between the most lateral ends of the greater trochanters; CD/AB, pelvic-trochanteric index.

Two experienced radiologists in musculoskeletal imaging gauged the digital X-rays using the Carestream Health software (Onex Corporation, Rochester, New York, USA), determined the horizontal lines between the most lateral part of the greater trochanters and the most lateral portions of iliac crests, and calculated the PTI, which is the ratio between these two measures. The femoral neck-shaft angle was also measured using radiographs, considering that the valgus angle decreases and the varus angle increases the horizontal femoral off-set and consequently the greater trochanteric prominence (Figure 1).

Agreement of the measurements between the radiologists was analyzed using three statistical techniques: intraclass correlation coefficient (ICC; values closer to 1 indicated higher agreement), percentage of agreement, and Kappa statistics. Clinical data were correlated with the PTI and femoral neck-shaft angle.

### Data analysis: general measures, comparisons, and associations

Absolute and percentile values of the control and case groups were obtained for qualitative (categorical) variables. For continuous quantitative variables, the mean standard deviation (SD), median, and interquartile range (IQR) were considered.

The case and control groups were compared using different variables and stratified analysis (Student’s t-test) for independent samples, Mann-Whitney test for medians of independent samples, Pearson’s chi-square for absolute values of ≥5, Fisher’s exact test for comparing absolute values <5, and Spearman’s rank correlation for continuous variables. Linear regressions were estimated to assess the independent associations, with robust SDs adjusted to minimize the effect of outliers. For all analyses, a significance level of 5% (*p*<0.05) was considered. When necessary, 95% reliability intervals are reported.

## RESULTS

### Reliability study for the radiographic measurements

For the agreement analysis between two radiologists, 51 X-rays (14 from the case group and 37 from the control group) were randomly selected using the Excel (Microsoft Corp). The mean and median age were 45.4 (SD=16.4) and 45 (IQR=27) years, respectively.

The agreement between the two radiologists in the distance between the greater trochanters (ICC=0.72) and femoral neck-shaft angle (ICC=0.73) was consistent. The agreement in measuring the distance between the iliac crest (ICC=0.99) and PTI (ICC=0.90) was almost perfect.

The agreement rate between the radiologists was 96.1% (49/51) in radiographic PTI measurements (Table 1), which was higher than the agreement that would occur at random (estimated 76.4%). The Kappa index was 0.834, which is considered an almost perfect agreement (reliability validity).

### Cases: description and comparison of diagnoses by age, age groups, and affected size

More than half of the patients were diagnosed with proximal iliotibial band syndrome (56%), followed by gluteal tendinopathy (35.7%). Patients with gluteal tendinopathy and concomitant proximal iliotibial band syndrome corresponded to 7.7% of the patients; one patient (0.6%) was diagnosed with external snapping hip.

When evaluating the symptomatic group, they can be analyzed according to the diagnosis of involvement (Table 2). There were no significant differences related to age, weight, or affected side.

### Case and control groups: correlation between measurements and age

**Figure 2:**
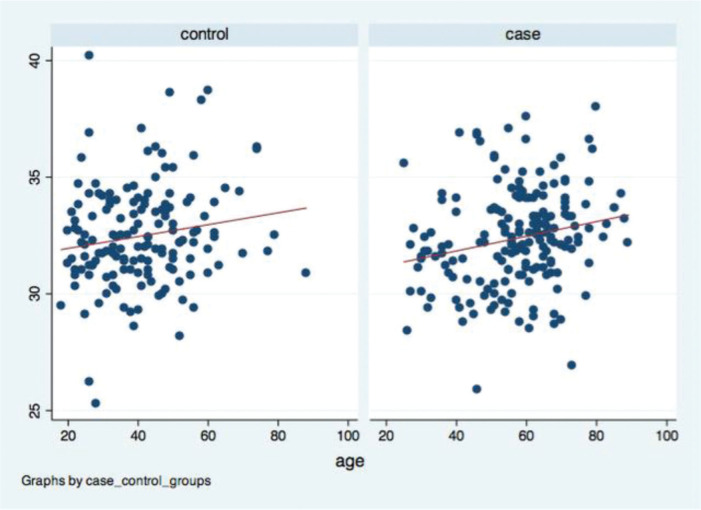
Dispersion graphic: age *versus* iliac crest distance.

**Figure 3:**
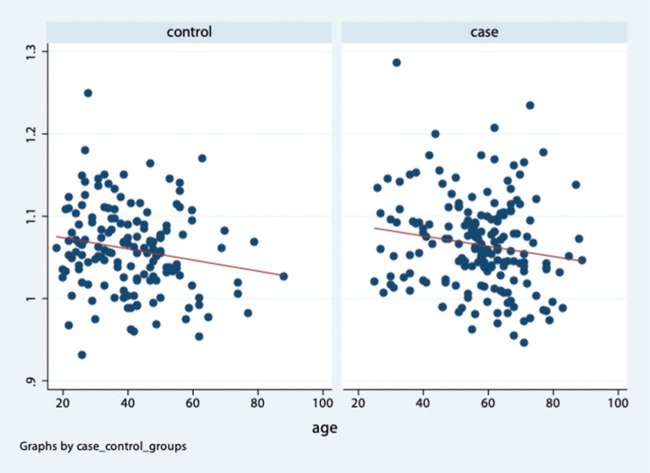
Dispersion graphic: age *versus* pelvic-trochanteric index.

No correlation was found between age and greater trochanter distance in both groups. The Spearman’s correlation coefficient was 0.052 (*p*=0.512) and 0.083 (*p*=0.267) in the case and control groups, respectively. A positive correlation was found between age and iliac crest distance in both groups (Figure 2). The Spearman’s correlation coefficient was 0.143 (*p*=0.082) in the control group and 0.239 in the case group (*p*=0.001). Meanwhile, a negative correlation was found between age and PTI. The Spearman’s correlation coefficient was −0.163 (*p*=0.046) in the control group and −0.151 (*p*=0.042) in the case group (Figure 3).

**Figure 4:**
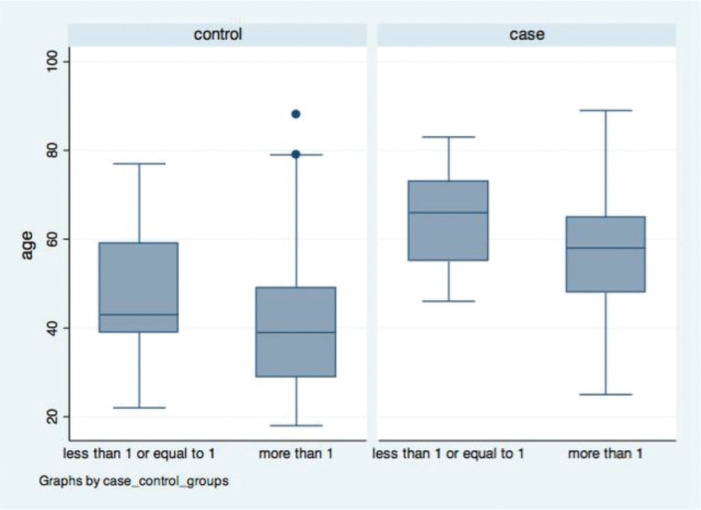
Box Plots: age *versus* pelvic-trochanteric index.

In the control group, the mean age of those with a PTI value of ≤1 and >1 was 46.5 (SD=14.2) and 40.3 (SD=13.6) years (*p*=0.048), respectively. In the case group, the corresponding mean age was 64.9 (SD=11.0) and 56.1 (SD=14.0) years (*p*=0.044). In both groups, the mean age was lower among those with a PTI value of >1 (Figure 4).

**Figure 5:**
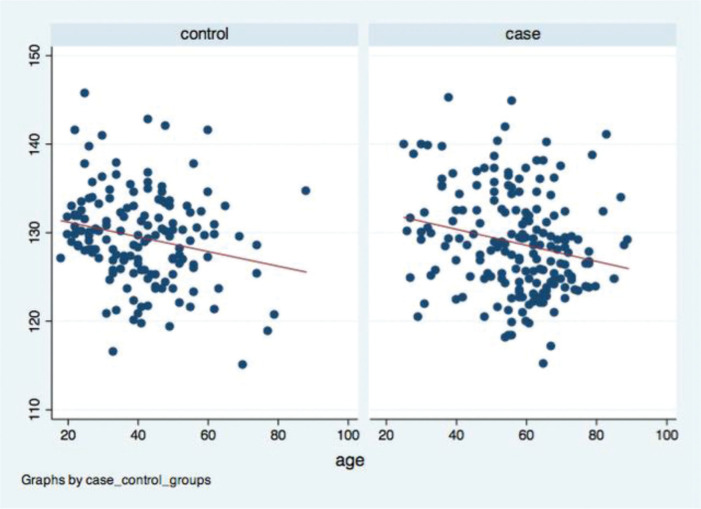
Dispersion graphic: age *versus* mean femoral neck-shaft angle.

A negative correlation was found between age and femoral neck-shaft angle in the case and control groups, with a significance level of 5%. The Spearman’s correlation coefficient was −0.199 (*p*=0.015) in the control and −0.228 (*p*=0.002) in the case groups (Figure 5).

### Correlation of case and control groups’ measurements with age groups

The mean distance between the greater trochanters for the asymptomatic group aged <40 years was 34.3 cm (SD=0.20) (Table 3). The mean distance of the greater trochanter was not significantly affected by age or the presence of symptoms.

In the asymptomatic group, the mean distance between the iliac crests for patients aged <40 years was 32.1 cm (SD=0.23). Among patients aged ≥60 years, the mean distance between the iliac crests increased by 1.1 cm (SD=0.34) (*p*=0.001) compared with the group aged <40 years (regardless of whether it was from the case or asymptomatic group). If they were 50-59 years old, the increase would be 0.65 cm (SD=0.35) (*p*=0.054).

The overall mean PTI value was 1.07 (SD=0.01), but in the case group, regardless of age, PTI value was ≥0.02 (SD=0.01) (*p*<0.05). Aging significantly reduced the PTI value (*p*<0.05), regardless of the group analyzed.

**Figure 6:**
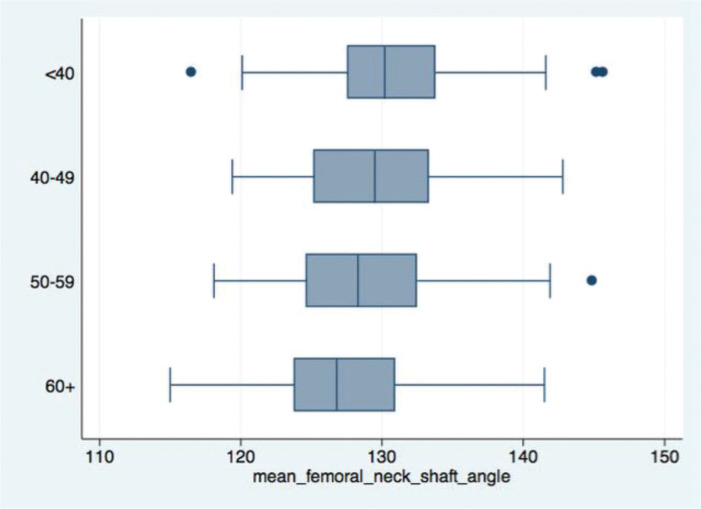
Box Plots: negative association between age and femoral neck-shaft angle.

Overall, the mean femoral neck-shaft angle was 130.6° (SD=0.59). A greater varus of the proximal femur was not observed in the case group. In addition, a progressive reduction in femoral neck-shaft angle was observed with aging, regardless of the group (Figure 6).

## DISCUSSION

Lateral hip pain or GTPS has been compared with shoulder rotator cuff disease (5,9). Despite being traditionally known as trochanteric bursitis, only 20% of patients present with bursa thickening (10-13).

According to Woyski et al. (14), gluteus medius tendinopathy is more common in women due to a smaller tendon insertion area and a shorter lever arm, thereby resulting in a smaller area to dissipate the tensile load and lower biomechanical efficiency, respectively. This results in a higher tensile load on the gluteus medius and minimus tendons.

Pelvic morphology has been hypothesized to be a risk factor for lateral hip pain (15). The greater trochanter is more prominent in the varus femoral neck, resulting in higher compression of the gluteus medius and minimus tendons by the iliotibial band (16). However, our study did not demonstrate an association between a larger varus of the proximal femur and GTPS.

According to Shbeeb et al. (3), GTPS affects people aged 40-60 years, as in our case group, and previous studies indicate that women over 40 years are associated with lateral hip pain (17,18). This is consistent with the present findings, which showed that more than 70% of symptomatic patients were over 50 years old.

It is necessary in a study that groups are comparable (homogeneous) in terms of demographic data such as age (19). In the present study, considering the heterogeneity in age distribution between groups, analyses were made by age range, which showed no association between age and distance between the greater trochanters, neither in the case nor in the control group.

In the present study, it was stipulated that a PTI value of >1 would be related to GTPS compared with that in asymptomatic individuals. Remarkably, it was identified that asymptomatic volunteers had a PTI value of >1, although they were lower than that of patients with GTPS.

When a multiple linear regression was performed, the mean PTI in the case group was greater than that in the control group (1.09 *versus* 1.07; *p=0,034*), regardless of age. This is in agreement with the findings of Viradia et al. (20), who observed an increased prominence of the greater trochanters in relation to the iliac wings in a group with trochanteric bursitis compared with in an asymptomatic control group. The present study also observed that PTI was negatively associated with aging (*p*<0.05), regardless of the group.

Few studies have evaluated skeleton enlargement after the age of 20 years, since it is considered that there is no further growth in height or width after skeletal maturity.

The present study showed a positive association between age and increase in the iliac crest distance in both groups, which agrees with the reports of Berger et al. (21) who found that the pelvis and the L4 vertebra had their width increased after skeletal maturity in both sexes. However, Huseynov et al. (22) showed that the pelvis narrows with aging, identifying that the female pelvis reaches its widest obstetrical morphology around the period of maximum fertility, thereby reducing its dimensions (22).

Although this is not a study that analyzes the variations in the pelvis of the same individual, we observed that the pelvis can have its dimensions changed with aging, since the distance between iliac crests also increased with aging. It was observed that in a patient aged ≥60 years, this distance was 1.1 cm greater (*p*=0.001) compared with that in a person aged <40 years. These data are consistent with the findings reported by Berger et al. (21), who found that the bone pelvis expands over 20 mm from ages 20 to 80 years (21).

The present study also showed that varus of the femoral neck is unrelated to GTPS and that the femoral neck-shaft angle progressively decreased with aging in both groups, which agrees with the findings of Pires et al. (23).

Considering that with aging, sarcopenia and fatty degeneration of the muscles with consequent loss of muscle strength occur, the progressive varus of the proximal femur with age would be a compensatory biomechanical alternative to increase the abductor lever arm and provide adequate tension of the gluteus medius and minimus to maintain the hip abductor muscle torque in balance with body weight torque.

When performing total hip arthroplasty, the components must be positioned to restore mechanical forces and range of motion (24). The hip abductor function is optimized by a slight increase in the horizontal femoral offset to improve the tension on the abductor muscles (25-27). In elderly patients who will undergo total hip arthroplasty, the extended (high) offset of the horizontal femoral component should be considered because of the compensatory varus of the proximal femur that occurs with age, as suggested by the present work. However, excessive horizontal femoral offset could result in greater tension on the footprint of the gluteus medius and minimus attached to the greater trochanter and consequent lateral hip pain (28,29).

This study presents the following limitations: male sex was not studied because of the disproportionality between sexes in the incidence of GTPS, which affects women preferably (4,18,). In addition, the use of CT scans would be more reliable than that of X-rays, but it carries the risk of exposure to higher levels of radiation.

## CONCLUSION

We found that the PTI was higher in female patients with GTPS. Therefore, PTI could be an important tool for the diagnosis of suspected GTPS or, at least, assist hip surgery for the prevention of GTPS in women with wide hips through physiotherapy.

The pelvic width tends to increase with age in symptomatic or asymptomatic patients, and the femoral neck-shaft angle does not differ between patients with and without GTPS; however, it decreases with age in symptomatic or asymptomatic individuals.

Therefore, the increase in pelvic width and decrease in the femoral neck-shaft angle can be interpreted as normal in aging women, with the potential to alter the biomechanics of the hips and pelvis.

## AUTHOR CONTRIBUTIONS

Santos LEN was responsible for data acquisition, analysis and interpretation, manuscript preparation, writing, and critical revision. Navarro TP was responsible for the study design and supervision. Machado CJ was responsible for the analysis, interpretation, and statistical analysis. Cabrita HABA was responsible for study conception and design, critical revision, provided substantial scientific and intellectual contributions to the study, and was responsible for the approval of the final version of the manuscript. Pires RE contributed substantially to the study design and critical revisions. Figueiredo LB was responsible for data acquisition and was responsible for the critical revision. Gurgel HMC was responsible for the study conception, design, and methodology of the manuscript. Pfeilsticker RM and Grandinetti H performed radiographic measurements. Souza AD contributed to the editing of the manuscript and critical revision. Andrade MAP was responsible for study supervision and critical revision, and provided substantial scientific and intellectual contributions to the study. All authors read and approved the final manuscript.

## Figures and Tables

**Table 1:** Agreement between Pelvic-Trochanteric Index measurements taken from the two radiologists.

Radiologist I			
**Radiologist II**	Lower or equal to 1	Higher than 1	Total
Lower or equal to 1	6 (11.7%)	1 (2.0%)	7 (13.7%)
Higher than 1	1 (2.0%)	43 (84.3%)	44 (86.3%)
Total	7 (13.7%)	44 (86.3%)	51 (100.0%)

Source: Prepared by the author.

Note. _absolute and percent numbers.

**Table 2:** Descriptive and comparative analysis of cases specifically by diagnosis.

Variables	Proximal iliotibial band syndrome (n=102; 100%)	Gluteal tendinopathy+proximal iliotibial band syndrome (n=14; 100%)	Gluteal tendinopathy (n=65; 100%)	*p*-value
Age (years)				
Mean (SD)	57.5 (14.1)	54,1 (11.3)	57.6 (14.4)	0.677
Median (IQR)	60 (18)	57.5 (18)	58 (17)	0.261
Minimum; Maximum	26; 87	33; 66	25; 89	
Age groups (years)				
Less than 40 (n; %)	15 (14.7)	3 (21.4)	7 (10.8)	0.423
40-49 (n; %)	10 (9.8)	1 (7.1)	9 (13.9)	
50-59 (n; %)	23 (22.6)	3 (21.4)	38 (35.4)	
60 or more (n; %)	54 (52.9)	7 (50.0)	26 (40.0)	
Affected side				
Bilateral (n; %)	35 (34.3)	4 (28.6)	13 (20.0)	0.264
Right (n; %)	35 (34.3)	7 (50.0)	27 (41.5)	
Left (n; %)	32 (31.4)	3 (21.4)	25 (38.5)	

Source: Prepared by the author.

Note: A 56-year-old patient, the only patient diagnosed with lateral hip snapping, was excluded.

SD, standard deviation; IQR, interquartile range; n, absolute number; *p*, significance level.

**Table 3:** Association of outcomes in cases and control groups *versus* age.

Variables	Asymptomatic group <40	Case (Ref: asymptomatic group)	40-49 years (Ref: <40)	50-59 years (Ref: <40)	60 years or older (Ref: <40)
Distance between greater trochanters (cm)	34.3 cm (SD=0.20)	-0.05 cm (SD=0.30) (-0.53; 0.43) *p*=0.824	0.05 cm (SD=0.30) (-0.57; 0.66) *p*=0.884	0.08 cm (SD=0.30) (-0.47; 0.64) *p*=0.767	0.33 cm (SD=0.30) (-0.27; 0.93) *p*=0.276
Distance between iliac crests (cm)	32.1 cm (SD=0.23)	-0.05 cm (SD=0.27) (-1.06; 0.05) *p*=0.076	0.61 cm (SD=0.35) (-0.12; 1.35) *p*=0.102	0.65 cm (SD=0.35) (-0.01; 1.31) *p*=0.054	1.1 cm (SD=0.34) (0.47; 1.75) *p*=0.001
Pelvic-Trochanteric Index	1.07 (SD=0.01)	0.02 (SD=0.01) (0.00; 0.03) *p*=0.034	-0.02 (SD=0.01) (-0.04; 0.00) *p*=0.033	-0.02 (SD=0.01) (-0.04; 0.00) *p*=0.023	-0.03 (SD=0.01) (-0.05; -0.01) *p*=0.005
Mean femoral neck-shaft angle (°)	130.6° (SD=0.59)	0.80° (SD=0.71) (-0.63; 2.23) *p*=0.272	-1.62° (SD=0.90) (-3.37; 0.12) *p*=0.067	-2.34° (SD=0.90) (-4,10; -0,58) *p*=0.002	-3.69° (SD=0.89) (-5.53; -1.84) *p*<0.001

Source: Prepared by the author.

Note: Ref, referential; cm, centimeter.
